# HGS Promotes Tumor Growth, Whereas the Coiled-Coil Domain and Its Oligopeptide of HGS Suppress It

**DOI:** 10.3390/ijms26020772

**Published:** 2025-01-17

**Authors:** Kiyoshi Ogura, Ikuo Kawashima, Kohji Kasahara

**Affiliations:** Biomembrane Group, Tokyo Metropolitan Institute of Medical Science, 6-1-2, Kamikitazawa, Setagaya-Ku, Tokyo 113-8613, Japan

**Keywords:** HGS, ESCRT, EMT, anchorage independent cell growth, tumor, inhibitor

## Abstract

We previously isolated a cDNA clone for galactosylceramide expression factor 1, which is the rat homologue of hepatocyte-growth-factor-regulated tyrosine kinase substrate (HGS) and induces galactosylceramide expression and morphological changes in COS-7 cells, and reported that overexpression of HGS induced morphological changes in canine kidney epithelial MDCK cells. HGS is a component of the endosomal sorting complexes required for transport machinery that mediates endosomal multivesicle body formation. In this study, the overexpression of HGS induced epithelial–mesenchymal transition and caused transformation in MDCK cells, whereas the overexpression of a coiled-coil domain of HGS inhibited induction of epithelial–mesenchymal transition by HGF stimulation. The overexpression of HGS in mouse melanoma B16 cells and human colorectal cancer COLO205 cells promoted cancer characteristic anchorage-independent cell growth ability and tumor growth, whereas the overexpression of the coiled-coil domain of HGS in these cells suppressed them. The oligopeptide OP12-462 constituting the coiled-coil domain suppressed the anchorage-independent cell growth ability and tumor growth of COLO205 cells. The coiled-coil domain of HGS and OP12-462 are novel tumor growth inhibitors that do not directly destroy cancer cells but rather inhibit only the anchorage-independent cell growth ability of cancer cells.

## 1. Introduction

Hepatocyte-growth-factor–regulated tyrosine kinase substrate (HGS) is identified and cloned as a 115 kDa protein that was rapidly tyrosine phosphorylated in hepatocyte growth factor (HGF)-treated B16 cells [1]. HGS knockout embryos show defects in ventral and anterior-to-caudal folding morphogenesis and are lethal at embryonic day 11, suggesting that HGS is essential for embryonic development [2]. HGS is a component of the endosomal sorting complexes required for transport (ESCRT) machinery [3,4,5,6,7]. The ESCRT machinery is an evolutionarily highly conserved process and is required for the multivesicular body pathway [8], autophagy [9,10], cytokinesis [11], and HIV budding [12]. The ESCRT is composed of five different ESCRT complexes: ESCRT-0, -I, -II, -III, and the Vps4 complex. HGS is composed of four domains: a zinc finger domain (HGS/Z), a proline-rich domain (HGS/P), a coiled-coil domain (HGS/C), and a proline–glutamine-rich domain (HGS/Q) [13]. HGS forms a heterodimer with a signaling adaptor molecule (STAM) via their coiled-coil domains to form ESCRT-0 [14]. The ESCRT-0, which binds ubiquitinated protein cargos and recruits them to ESCRT-I, is the first step in the ESCRT machinery. The ESCRT machinery mediates signal transductions through endosomal multivesicle formation and contributes to anchorage-independent growth, a hallmark of cancer characteristics [15,16,17].

During normal development, tissue reorganization, wound healing, carcinogenesis, and metastasis, epithelial cells lose transient or stable epithelial polarity, including an intercellular adhesion, and acquire mesenchymal phenotypes, including a fibroblastic spindle-shape and an increased motility. The process of these phenotypical changes has been termed epithelial–mesenchymal transition (EMT) [18,19]. EMT is a highly conserved and fundamental process that governs morphogenesis in multicellular organisms [20] and is integral in development, wound healing, and stem cell behavior and contributes pathologically to fibrosis and cancer progression [18,19,21]. E-cadherin is one of the marker proteins of the epithelial phenotype, and loss of E-cadherin expression is involved in EMT. E-cadherin mediates homophilic interactions in its extracellular region and connects to actin indirectly via α-catenin and β-catenin in the cytoplasm. E-cadherin induces the formation of stable cell–cell contacts and the development of adherent junctions and is required for the maintenance of stable junctions. β-Catenin is involved in the transduction of the Wnt signaling pathway, which is important for early embryonic patterning, cell polarity, and tumor cell metastasis [22]. In the Wnt signaling pathway, nuclear translocation of β-catenin activates the TCF/LEF transcription factor, promoting two critical malignant processes: invasion and metastasis [23]. Several transcription factors have been implicated in EMT, including zinc-finger proteins in the Snail/Slug family [24] and the basic helix–loop–helix E47 factor [25]. The activities of these transcription factors are regulated by signal-transductions from several pathways including HGF, TGF-β [20], and Wnt [21]. Madin-Darby canine kidney (MDCK) cells exhibit a typical epithelial-like shape, forming tight monolayers with junctional complexes, and maintain a flat confluent state through contact inhibition. MDCK cells undergo EMT by HGF and TGF-β stimulation and exhibit enhanced cell motility and a ruffling morphology [21,26]. This in vitro transition is one of the model systems for EMT and has been used to study the mechanisms of EMT.

We previously isolated a cDNA clone for galactosylceramide expression factor 1, which is a rat homologue of hepatocyte-growth-factor–regulated tyrosine kinase substrate (HGS) that induces galactosylceramide expression and morphological changes in COS-7 cells [13]. To elucidate the function of HGS in inducing cellular morphological changes, We established MDCK cells overexpressing HGS (MDCK/HGS), which showed fibroblast-like morphology even without HGF stimulation [27] and were induced to undergo myogenesis for long-term culture [28]. Studies on HGS deletion mutants showed that HGS/C and HGS/Q domain were essential for morphological changes in MDCK cells. The morphological changes in MDCK/HGS cells suggest that HGS is involved in HGF and TGF-β signal transduction and EMT induction. In this study, we demonstrated that HGS promoted EMT and cancer characteristics in MDCK cells, mouse melanoma B16 cells, and human colorectal COLO 205 cells, whereas HGS/C and an oligopeptide constituting HGS/C suppressed them.

## 2. Results

### 2.1. HGS Induces EMT in MDCK Cells

**Figure 1:**
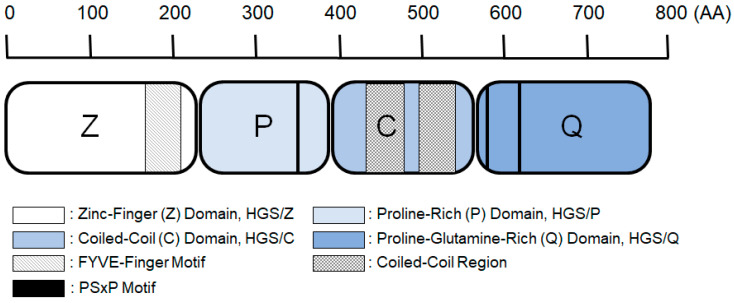
Schematic diagram showing the structure of HGS. Modified from Reference [13].

**Figure 2:**
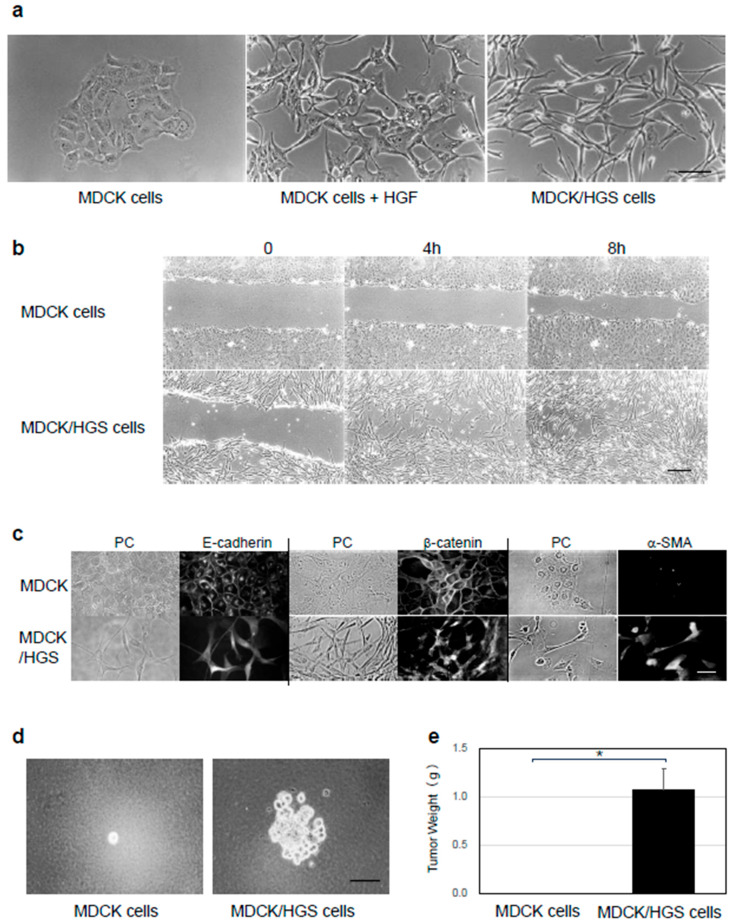
Characterization of MDCK/HGS cells. (**a**) Colony and cell morphology of MDCK and MDCK/HGS cells. MDCK cells were incubated with or without HGF (50 ng/mL) for 24 h. Morphological changes are induced in MDCK cells by HGF stimulation. MDCK/HGS cells were changed in morphology without HGF stimulation. The bar represents 100 μm. (**b**) Scratch assay of MDCK, and MDCK/HGS cells. MDCK, and MDCK/HGS cells at 0, 4 h and 8 h after scratching. The bar represents 100 μm. (**c**) Localization and expression of marker proteins. MDCK and MDCK/HGS cells were immunostained with monoclonal antibodies against the marker proteins, E-cadherin, β-catenin, and α-smooth muscle actin. The bar represents 50 μm. (**d**) Colony formation of MDCK and MDCK/HGS in soft agar medium. MDCK and MDCK/HGS cells were cultured in soft agar medium from a single cell for 14 days. The bar represents 100 μm. (**e**) Tumor formation of MDCK and MDCK/HGS cells in nude mice. MDCK or MDCK/HGS cells (1 × 10^6^ cells) were inoculated subcutaneously into the flank region of nude mice (*n* = 6), and tumors were harvested and weighed 28 days later. MDCK/HGS cells formed subcutaneous tumors in nude mice. The asterisk indicates statistical significance.

Epithelial–mesenchymal transition (EMT) is a phenotypic change in which epithelial cells transiently or stably lose epithelial polarity, including cell–cell adhesion, and acquire a mesenchymal phenotype, including a spindle shape and increased motility. HGF induces EMT in MDCK cells, resulting in a change in morphological cell shape [29]. Morphological changes in MDCK cells by HGF stimulation is one model system for EMT. We reported that cloning cDNA of galactosylceramide expression factor-1 (Figure 1) [13] is a rat orthologue of HGS [1] and overexpression of HGS induced MDCK cells to change to a fibroblast-like spindle morphology in the absence of HGF stimulation. (Figure 2a) [27].

In this study, we first investigated the cellular characteristics of HGS-overexpressing MDCK (MDCK/HGS) cells to clarify the involvement of HGS in EMT. MDCK/HGS cells were examined for their migration ability by scratch assay, the localization of epithelial and mesenchymal characteristic proteins by immunostaining, and their anchorage-independent growth ability by proliferating them in soft agar medium and subcutaneous tumors in nude mice. Mesenchymal cells have the capability of high migratory property. To examine the migratory property of MDCK/GEF-1 cells, we first employed a scratch assay (Figure 2b). All or most of the wound surface was colonized by MDCK/HGS cells at 8 h after the wound was made, whereas, at the same time, MDCK cells had not colonized half the area of the wound surface yet (Figure 2b). The scratch assay showed that MDCK/HGS cells had increased cell mobility. The localization and expression of marker proteins were next observed by immunofluorescence analysis (Figure 2c). E-cadherin and β-catenin, epithelial cellular marker proteins, were observed in the cytosol and the nucleus of MDCK/HGS cells, whereas they were observed on the plasma membrane of MDCK cells (Figure 2c) by immunofluorescence analysis. α-Smooth muscle actin (α-SMA), a mesenchymal cellular marker protein, was observed in the cytosol in MDCK/HGS cells but not in MDCK cells. These findings indicated that MDCK/HGS cells were mesenchymal-like cells but not epithelial-like cells. MDCK/HGS cells formed colonies in soft agar medium (Figure 2d) and subcutaneous tumors in nude mice (Figure 2e), demonstrating a cancer characteristic. These results suggest that the overexpression of HGS induces EMT and tumorigenicity in MDCK cells.

### 2.2. HGS/CQ Induces EMT, Whereas HGS/C Inhibits EMT

The rat HGS molecule is composed of four domains: a zinc finger domain (HGS/Z, 1–233) with zinc fingers, a proline-rich domain (HGS/P, 234–390) with a proline content of 14.3%, a coiled-coil domain (HGS/C, 391–559) with a coiled-coil region, and a proline–glutamine-rich domain (HGS/Q, 560–771) with a proline content of 20.3% and a glutamine content of 13.4% (Figure 1) [13]. We previously established stable MDCK mutant cells overexpressing HGS deletion mutants [27]. To study the involvement of HGS in EMT, we investigated the morphological changes in the MDCK mutant cells with or without HGF stimulation (Table 1). As a result, we found that the overexpression of HGS/ZPCQ (native HGS), HGS/PCQ, and HGS/CQ induced the morphological changes in the MDCK/ZPCQ, MDCK/PCQ, and MDCK/CQ cells, respectively, without HGF stimulation. These HGS mutants possessed the ability to induce EMT. The overexpression of HGS/Z, HGS/ZP, HGS/P, and HGS/Q induced the morphological changes in the MDCK/Z, MDCK/ZP, MDCK/P, and MDCK/Q cells, respectively, with HGF stimulation but not in absence of HGF stimulation, suggesting that these HGS mutants have no effect on EMT induction. HGS mutants containing HGS/CQ could induce EMT in MDCK cells without HGF stimulation, suggesting that HGS/CQ transduces downstream signals of HGF stimulation even in the absence of HGF stimulation. The overexpression of HGS/ZPC, HGS/PC, and HGS/C did not induce any morphological changes in MDCK/ZPC, MDCK/PC, and MDCK/C cells, respectively, with or without HGF stimulation, suggesting that these domains inhibit the transduction of downstream signals of HGF stimulation, thereby inhibiting EMT. HGS/C with HGS/Q (HGS/CQ) was essential for EMT induction in MDCK cells, whereas HGS/C without HGS/Q (HGS/C) inhibited EMT induction. In MDCK/HGS and MDCK/C cells, we measured the transcriptional activity of SMAD transcription factors downstream of the TGF-β signaling pathway, which is involved in EMT and tumorigenicity. We found that in MDCK cells, TGF-β stimulation increased the transcriptional activity of SMAD elements by 11.1-fold, whereas in MDCK/HGS cells overexpressing HGS, the SMAD transcriptional activity increased by 563-fold without TGF-β stimulation and further increased by 3680-fold with TGF-β stimulation (Table 2). HGS always transduces downstream signals of TGF-β stimulation even in the absence of TGF-β stimulation. However, in MDCK/C cells overexpressing only HGS/C, the SMAD transcriptional activity was equivalent to that in MDCK cells without TGF-β stimulation and did not increase upon TGF-β stimulation, suggesting that HGS/C inhibits the transduction of downstream signals of TGF-β stimulation and has dominant-negative effects on signal transductions. These results suggested that HGS induced EMT and tumorigenicity via TGF-β signal transduction in MDCK cells, and HGS/C inhibited the signal transduction.

### 2.3. HGS Promotes Cancer Characteristics, Whereas HGS/C Suppresses Them in Mouse Melanoma B16 Cells

**Figure 3:**
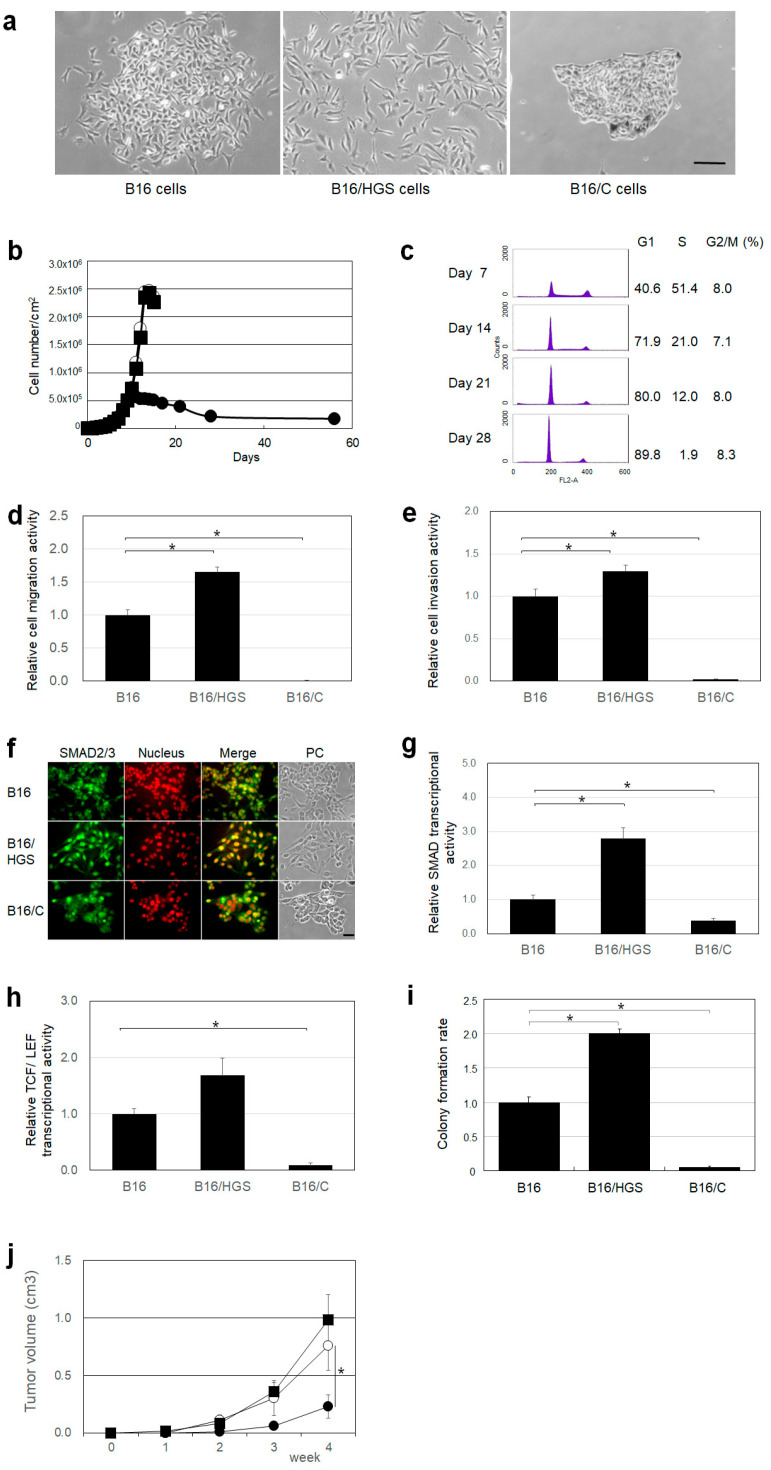
Characterization of B16/HGS and B16/C cells. (**a**) Colony morphology of B16 cells, B16/HGS, and B16/C cells. B16, B16/HGS, and B16/C cells were cultured from single cells for 14 days. The bar represents 100 μm. (**b**) Cell growth of B16 cells, B16/HGS cells, and B16/C cells. B16, B16/HGS, and B16/C cells were seeded at a density of 5.0 × 10^3^ cells/cm^2^ and cultured for a long period. B16 cells, (○). B16/HGS cells, (■). B16/C cells, (●). (**c**) Culture days and cell cycle of B16/C cells. B16/C cells were cultured for a long period and their cell cycle was analyzed on a FACScan flow cytometer (BD Biosciences, Franklin Lakes, NJ, USA) using BD Cycle Test Plus DNA Reagent Kit (BD Biosciences). (**d**) Cell migration activity of B16, B16/HGS, and B16/C cells. Cell motility was assayed using biocoat culture inserts. The asterisks indicate statistical significance. (**e**) Cell invasion activity of B16, B16/HGS, and B16/C cells. Cell invasive activity was assayed using Matrigel Inversion chambers. The asterisks indicate statistical significance. (**f**) Localization of SMAD2/3 in B16, B16/HGS, and B16/C cells. B16, B16/HGS, and B16/C cells were immunostained with a monoclonal antibody against SMAD2/3. The cell nuclei were subsequently stained with propidium iodide. The bar represents 50 μm. (**g**) Transcriptional activity of SMAD of B16, B16/HGS, and B16/C cells. Smad RE-TK hRluc, or TK hRluc, and SV-luc reporter gene plasmids were transiently transfected into B16, B16/HGS, and B16/C cells. Transcriptional activities of SMAD were calculated from Renilla luciferase activity and firefly luciferase activity. The asterisks indicate statistical significance. (**h**) Transcriptional activity of TCF/LEF of B16, B16/HGS, and B16/C cells. TCF/LEF RE-TK hRluc, or TK hRluc, and SV-luc reporter gene plasmid were transiently transfected into B16, B16/HGS, and B16/C cells. Transcriptional activities of TCF/LEF were calculated from Renilla luciferase activity and firefly luciferase activity. The asterisk indicates statistical significance. (**i**) Colony formation of B16 cells, B16/HGS cells, and B16/C cells in soft agar medium. B16, B16/HGS, and B16/C cells were cultured in soft agar medium from a single cell for 14 days. The number of colonies formed was counted. The asterisks indicate statistical significance. (**j**) Tumor formation of B16 cells, B16/HGS cells, and B16/C cells in C57BL/6 mice. B16, B16/HGS, and B16/C cells (1 × 10^6^ cells) were inoculated subcutaneously into the flank region of C57BL/6 mice (*n* = 5), and tumor formation was monitored for 4 weeks after the inoculation by measuring the width and length of the tumors. B16 cells, (○). B16/HGS cells, (■). B16/C cells, (●). The asterisk indicates statistical significance.

Because EMT occurs not only in epithelial cells but also in neural crest cells, we studied the effects of HGS and HGS/C on mouse melanoma B16 cells, which are derived from neural-crest-derived cells. B16 cells have been widely used as target cells in an animal model of cancer invasion and metastasis both in vitro and in vivo. We established stable B16 cells overexpressing HGS (B16/HGS cells) or the HGS/C (B16/C cells) and investigated the colony formation, proliferation, motility, invasiveness, signal transductions, and tumor formation of the B16 mutant cells. There was a difference in colony formation between B16/HGS and B16/C cells (Figure 3a). B16/HGS cells were dispersed, whereas B16/C cells formed tightly packed colonies, suggesting that B16/HGS cells are highly motile and B16/C cells are less motile (Figure 3a). B16/HGS cells grew to a high density similar to B16 cells. The growth of B16/C cells was inhibited upon contact with each other (i.e., contact inhibition) at a density over 5 × 10^5^ cells/cm^2^ (Figure 3b). The contact inhibition caused B16/C cells to arrest in the G1 phase of the cell cycle (Figure 3c). In vitro, B16/HGS cells showed a 1.7-fold increase in cell migration activity and a 1.3-fold increase in cell invasion activity than B16 cells, whereas B16/C cells almost lost both cell motility and invasiveness (Figure 3d,e). We investigated the subcellular localization of SMAD2/3 in B16, B16/HGS, and B16/C cells (Figure 3f). Many SMAD2/3 nuclear localizations were observed in B16 cells. In B16/HGS cells, most of SMAD2/3 was localized in the nucleus. In B16/C cells, SMAD2/3 was less localized in the nucleus than in B16 cells. Next, we measured SMAD and TCF/LEF transcriptional activities in B16, B16/HGS, and B16/C cells to investigate TGF-β and Wnt/β-catenin signal transduction, respectively. In B16/HGS cells, the transcriptional activities of SMAD and TCF/LEF were increased by 2.8- and 1.7-fold compared with those of B16 cells, whereas in B16/C cells, the transcriptional activities of SMAD and TCF/LEF were decreased by 0.39- and 0.087-fold, respectively (Figure 3g,h). These results indicate that TGF-β-SMAD signal transduction is increased in B16/HGS cells and decreased in B16/C cells compared to B16 cells. The number of colonies in soft agar medium was increased 2.0-fold in B16/HGS cells and decreased 0.02-fold in B16/C cells compared with those of B16 cells (Figure 3i). The estimated mean tumor volume of B16/HGS cell tumors was increased by 1.5-fold compared with that of HGS cell tumors, whereas the tumor volume of B16/C cell tumors was reduced by 0.2-fold compared with that of HGS cell tumors (Figure 3j). The anchorage-independent growth ability and tumorigenicity of B16/HGS cells in vitro and in vivo, respectively, were increased compared with those of B16 cells but were decreased in B16/C cells. These results suggest that HGS promotes cancer characteristics, such as cell motility, invasiveness, and the anchorage-independent growth ability, of B16 cells in vitro and in vivo, whereas HGS/C suppresses them.

### 2.4. HGS/C Suppressed Tumorigenicity in Human Colorectal Cancer COLO205 Cells

**Figure 4:**
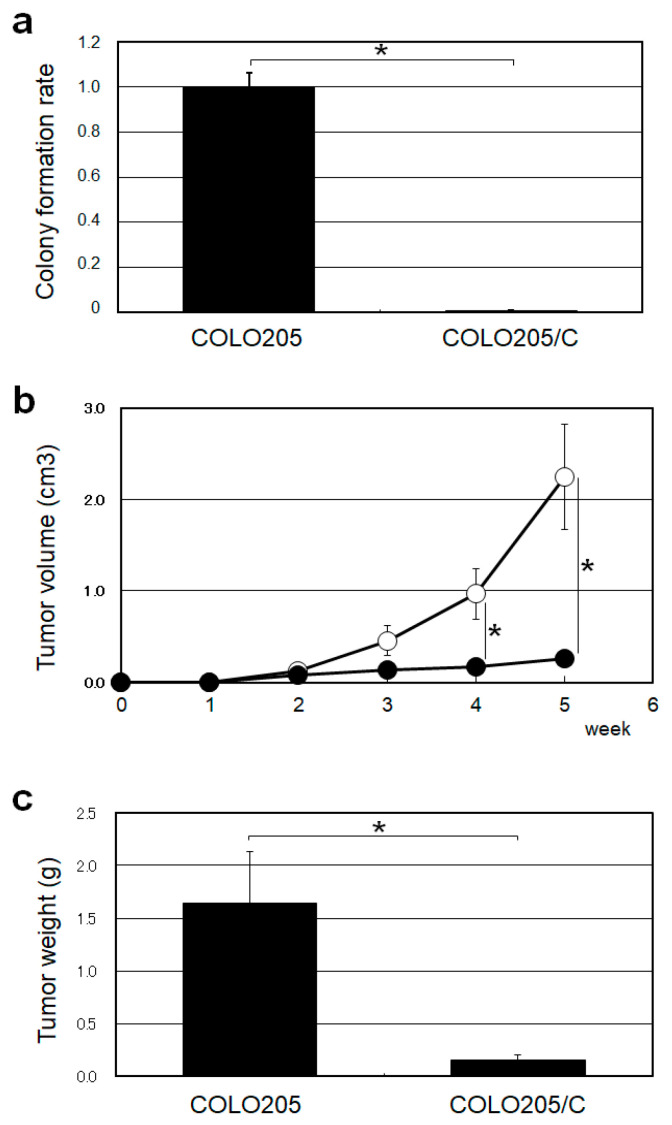
Characterization of COLO205/C cells. (**a**) Colony formation of COLO205 and COLO205/C cells in soft agar medium. COLO205 and COLO205/C cells were cultured in soft agar medium from a single cell for 14 days. The number of colonies formed was counted. The asterisk indicates statistical significance. (**b**) Tumor growth of COLO205 and COLO205 cells in nude mice. COLO205 and COLO205/C cells (1 × 10^6^ cells) were inoculated subcutaneously into the flank region of nude mice (*n* = 5), and tumor formation was monitored for 5 weeks by measuring the width and length of the tumors. COLO25 cells, (○). COLO205/C cells, (●). The asterisks indicate statistical significance. (**c**) Tumor weight of COLO205 and COLO205/C cells in nude mice. Five weeks after inoculation, the COLO205 and COLO205/C cells formed were harvested and their wet weights were measured. The asterisk indicates statistical significance.

We next studied the tumorigenic effects of HGS/C on human cancer cells. We established stable human colorectal cancer COLO205 cells overexpressing HGS/C (COLO205/C cells) and investigated their colony formation in soft agar medium and tumor formation in nude mice. In soft agar medium, the colony-forming ability of COLO205/C cells was almost lost (Figure 4a). In nude mice, the estimated tumor volume and the wet weight of COLO205/C cell tumors were reduced to one-tenth of that of COLO205 cell tumors 5 weeks after inoculation (Figure 4b,c). The tumorigenicity of subcutaneously implanted COLO205/C cells was significantly reduced compared with that of COLO205 cells. The overexpression of the HGS/C strongly suppressed the anchorage-independent growth ability of COLO205 cells and tumorigenicity. These findings strongly suggest that HGS/C suppresses tumorigenicity of cancer cells.

### 2.5. Oligopeptides Constituting HGS/C

**Figure 5:**
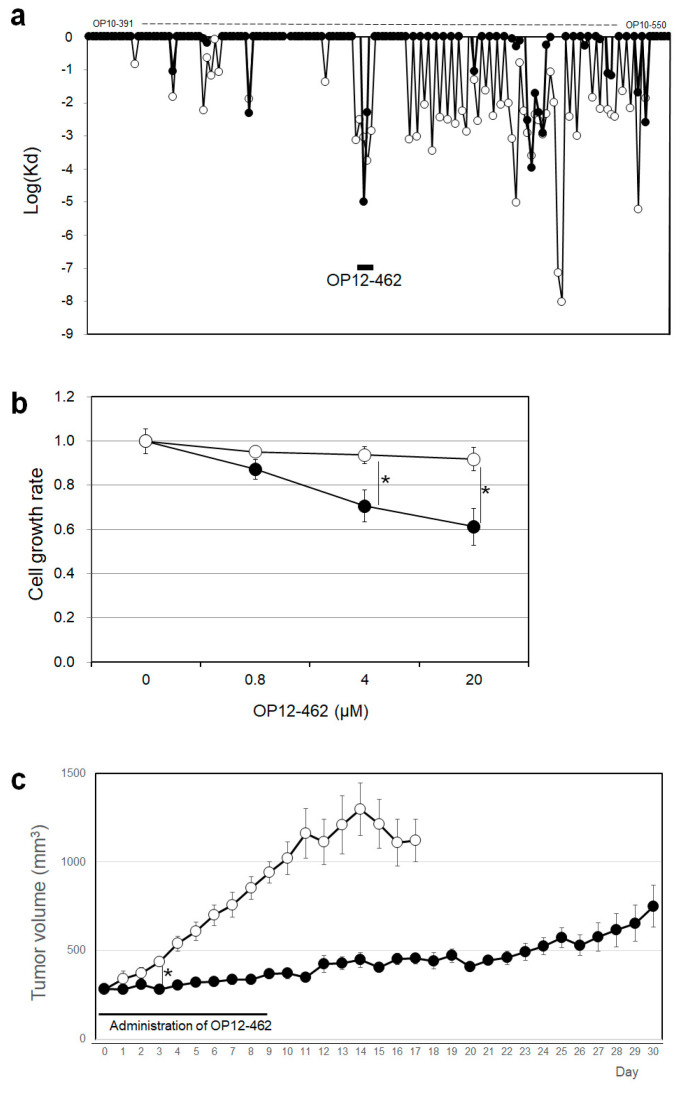
Characterization of OP12-462. (**a**) Analysis of the interaction of HGS/C oligopeptides with HGS and STAM by surface plasmon resonance. The dissociation rate constants (Kd) between HGS/C oligopeptides and immobilized proteins, HGS and STAM, were measured. HGS, (○). STAM, (●). (**b**) Effects of OP12-462 on cell growth of COLO205/C cells in normal adhesion plates and ULAS plates. COLO205/C cells were cultured in normal adhesion plates and ULAS plates, and the effects of OP12-462 on cell proliferation were measured. Normal adhesion plate, (○). ULAS plate, (●). The asterisks indicate statistical significance. (**c**) Tumor growth inhibition of COLO205 cells by tail vein administration of OP12-462. COLO205 cells were inoculated subcutaneously into the flank region of nude mice (*n* = 5). The day when the tumor volume exceeded 250 mm^3^ was set as Day 0. Oligopeptide OP12-462 (50 mg/kg body weight) was administered as 0.2 mL PBS solution once daily into the tail vein for 10 days from day 0 to day 9. The tumor sizes of mice that died after Day 15 in the vehicle group were excluded from the calculation. The difference in tumor volume between the OP12-462 group and the vehicle group was statistically significant from the third day onwards. vehicle, (○); OP12-462 (●). The asterisks indicate statistical significance.

The HGS/C showed strong inhibitory activity against the anchorage-independent growth ability of cancer cells, which is involved in tumor growth. Next, we studied whether the oligopeptides constituting HGS/C can also inhibit the anchorage-independent growth ability of cancer cells. HGS and STAM heterodimerize through their respective coiled-coil domain (HGS/C and STAN/C) to form ESCRT-0, the first step in the ESCORT machinery involved in signal transduction. To compensate for the dominant-negative effect of HGS/C on tumor growth, it was necessary to search for oligopeptides that can tightly associate to HGS, STAM, or both HGS and STAM and inhibit the dimerization of HGS and STAM. First, we prepared an oligopeptide library of HGS/C amino acid sequences. The HGS/C oligopeptides in this library have a length of 10 amino acid residues and have the amino acid sequence of HGS/C shifted by one amino acid each in the order from the N-terminus (OP10-391 (amino acid number of HGS) to the C-terminus (OP10-541); that is, sequences overlap by nine amino acid residues, thereby covering the amino acid sequence of the HGS/C domain. The HGS/C oligopeptides that interact with HGS and STAM were screened using the ProteOn XPR36 intermolecular interaction array system (Bio-Rad). We measured the dissociation rate constants (Kd) of the HGS/C oligopeptides for each of HGS and STAM (Figure 5a). As a result, OP10-462–OP10-464 and OP10-463 were found to interact strongly with HGS and STAM, respectively. The oligopeptide OP12-462 (ERRLYYEGLQDK), consisting of 12 amino acid residues, which covers the sequence of OP10-462–OP10-464 by extending two amino acid residues at the C-terminus of OP10-462, was selected as a candidate oligopeptide.

### 2.6. OP12-462 Inhibits Anchorage-Independent Growth

The effect of OP12-462 on anchorage-independent growth was examined; normal cells can proliferate in an anchorage-dependent manner but cannot in an anchorage-independent manner, whereas cancer cells can both proliferate in the anchorage-independent manner and form tumors. In normal (adhesion) plates, cancer cells adhere to the surface of plates and proliferate in the anchorage-dependent manner, so the anchorage-independent growth ability of the cancer cells cannot be observed. In ultra-low attachment surface (ULAS) plates, the cancer cells cannot adhere to the surface nor proliferate in the anchorage-dependent manner but can proliferate in the anchorage-independent manner. By measuring the proliferation ability of cancer cells on the ULAS plates, we can determine the anchorage-independent growth ability of the cells. OP12-462 inhibited the anchorage-independent growth activity of COLO 205 cells (Figure 5b) in a concentration-dependent manner in the ULAS plates. OP12-462 markedly inhibited the anchorage-independent growth of the COLO205 cancer cells but not the anchorage-dependent growth. OP12-462 did not inhibit the anchorage-dependent growth of MDCK normal cells at all in the normal (adhesion) plates. Next, the effect of OP12-462 on tumor growth was examined. COLO 205 cells were inoculated subcutaneously into the back of BALB/cSlc-nu/nu nude mice. The day when the tumor volume exceeded 250 mm^3^ was set as day 0, and OP12-462 (50 mg/kg body weight) in 0.2 mL of PBS solution or vehicle (PBS) was administered once a day into the tail vein for 10 days from day 0 to day 9. OP12-462 significantly suppressed the growth of the tumor not only during the administration period but also for more than two weeks after the administration had ended (Figure 5c). Ten days after administration, the changes in body weight of each mouse were found −0.4% to +2% with no change in the average body weight. No acute toxicity due to the administration of OP12-462 was observed.

## 3. Discussion

In this study, we demonstrated that ESCRT and EMT are closely related. The study of HGS overexpression in MDCK cells demonstrated that HGS induced EMT and tumorigenicity in MDCK cells, as shown by the basis of the following findings: (1) HGS induced morphological changes from epithelial cells to fibroblast-like cells; (2) HGS promoted cell motility and invasion activity; (3) HGS induced localization shifts of E-cadherin and β-catenin from the plasma membrane to the cytoplasm and nucleus and expression of α-smooth muscle actin, a mesenchymal protein; (4) HGS induced the colony-formation ability in soft agar medium and the tumor-growth ability in mice, suggesting the induction for carcinogenic transformation. The study of HGS deletion mutants in MDCK cells demonstrated that HGS/CQ was essential for HGF and TGF-β-SMAD signal transduction and HGS/C inhibited their signal transductions: (1) HGS deletion mutants containing HGS/CQ without HGF stimulation induced morphological changes; (2) HGS deletion mutants containing HGS/C and lacking HGS/Q with HGF stimulation inhibited morphological changes; (3) HGS promoted TGF-β-SMAD signal transduction in the SMAD elements assay, whereas HGS/C inhibited it. This finding suggests that HGS deletion mutants containing HGS/CQ, especially HGS/CQ, can transduce the signals, but HGS deletion mutants containing HGS/C and lacking HGS/Q, especially HGS/C, can inhibit them. HGS/C associates with STAM to form ESCRT-0 and recruits ubiquitinated protein cargos. HGS/Q contains two sites of the PSxP motif that recruits ESCRT-I. HGS/C and HGS/Q were essential for signal transduction by HGS. ESCRT-0 lacking HGS/Q cannot recruit ESCRT-I nor transfer the ubiquitinated protein cargos to the ESCRT machinery, resulting in failure to transduce signals.

The study of HGS and HGS/C overexpression in cancer cells demonstrated that HGS promoted tumorigenicity in B16 cells and COLO205 cells, whereas HGS/C suppressed it: (1) HGS promoted the TGF-β-SMAD and Wnt-β-catenin signal transduction, whereas HGS/C suppressed them; (2) HGS promoted the colony-formation ability in soft agar medium and promoted their tumor-growth ability in mice, whereas HGS/C suppressed them.

The study of the oligopeptide OP12-462 demonstrated that the inhibited anchorage-independent growth did not affect anchorage-dependent growth and significantly suppressed the tumor growth of COLO205 cells without any loss of mouse weight by tail vein administration.

The results of our study suggest that HGS promotes TGF-β-SMAD and Wnt-β-catenin signal transduction through the ESCRT machinery, thereby promoting the anchorage-independent growth of cancer cells, whereas HGS/C and HGS/C oligopeptide OP12-462 suppressed them. These results suggest that HGS/C lacked HGS/Q and could bind STAM and the ubiquitinated protein cargos, whereas it could not recruit ESCRT-I and transfer the ubiquitinated protein cargos to ESCRT-I.

Several studies have been conducted on the involvement of HGS and ESCRT in tumorigenesis. Toyoshima et al. reported that the down-regulation of HGS by siRNA reduced the tumorigenic potential by inhibiting the colony formation and metastasis of HeLa cells due to the increased E-cadherin expression level [30]. Mattissek and Teis also reported the roles of ESCRT in tumorigenesis [31].

HGS/C and oligopeptides constituting HGS/C, including OP12-462, might be new tumor growth inhibitors that suppress tumor growth by inhibiting the anchorage-independent growth ability of cancer cells rather than directly destroying cancer cells. Overexpression of HGS/C did not inhibit the anchorage-dependent growth of MDCK, B16, or COLO205 cells. OP12-462 shows no acute toxicity to normal or cancer cells in vitro. Furthermore, the administration of OP12-462 at 50 mg/kg for 10 consecutive days did not result in weight loss in nude mice. These findings suggest that OP12-462 may be used as an antitumor medicine with minimal side effects on normal cells in the body and can be administered to patients with reduced physical strength. HGS/C and the OP12-462 are novel inhibitors of TGF-β-SMAD and Wnt-β-catenin signal transduction. They not only have a novel tumor growth suppression effect but also have the potential to become novel medicines for diseases, including various fibrosis diseases, whose symptoms worsen owing to the enhancement of these signal transduction pathways. Potential drug targets of EMT in metastasis are now in development. HGS and HGS/C are an inducer and an inhibitor of EMT and tumorigenesis. Therefore, HGS and HGS/C could be good targets of EMT-blocking drugs for clinical application.

Inhibitors of HGS expression also have potential as HGS-targeting drugs in addition to inhibitors of HGS function. Expression inhibitors include nucleic acids used for RNA interference (microRNA, shRNA, siRNA), antisense nucleic acids, decoy nucleic acids, or aptamers. These expression inhibitors are capable of inhibiting HGS expression. The siRNA and the shRNA for HGS showed suppressive effects on tumor growth in vivo (unpublished data). In recent years, there has been a rapid advancement in the field of technology for chemically degrading target proteins in vivo. Proteolysis-targeting-chimera (PROTAC) exploits the ubiquitin–proteasome system degradation pathway to achieve therapeutic effects by down-regulating protein levels rather than inhibiting protein function [32]. Therefore, HGS can be a potential target for the PROTEC.

We are currently investigating small-molecule compounds targeting HGS, STAM, and ESCRT-0 as antitumor agents. Low-molecular-weight compounds that inhibit the dimerization of HGS/C and STAM/C, such as HGS/C and OP12-462, are expected to exhibit tumor growth inhibitory effects. We have constructed a high-throughput split-GFP assay system [33] to observe the dimerization of HGS/C and STAM/C, and we are screening for small-molecule compounds with similar functions to OP12-462.

## 4. Materials and Methods

### 4.1. Antibodies and Reagents

Anti-E-cadherin and anti-β-catenin antibody from BD Transduction Laboratories (BD Bioscience, San Jose, CA, USA), α-smooth muscle actin antibody from Oncogene Research Products (Boston, MA, USA), and Alexa Fluor 488–conjugated goat F (ab’)_2_ fragment against mouse IgG from Thermo Fisher Scientific (Waltham, MA, USA) were purchased. Cell Counting Kit-8 was purchased from Dotjindo (Kumamoto, Japan).

### 4.2. Proteins

GST-HGS, GST-STAM, and GST were purchased from Abnova (Taipei City, Taiwan).

### 4.3. Oligopeptides

HGS/C constituent oligopeptides were synthesized by entrusting their synthesis to GL Biochem (Shanghai, China) Ltd., China.

### 4.4. Cells

Madin-Darby canine kidney (MDCK) cells and Human colorectal cancer COLO205 cells were purchased from RIKEN (Tsukuba, Japan) and ATCC (Manassas, VA, USA), respectively. Mouse melanoma B16 cells, which were derived from C57BL/6 mouse, were obtained from the Cell Resource Center for Biomedical Research, Tohoku University (Sendai, Japan). The cells were cultured in RPMI-1640 Medium (Gibco, Waltham, MA USA) supplemented with 10% fetal bovine serum (HyClone), penicillin, and streptomycin (Gibco, Waltham, MA, USA) at 37 °C in 5% CO_2_.

### 4.5. Mice

C57BL/6 (C57BL/6NCrSlc) and Nude mice (BALB/cSlc-*nu/nu*) were purchased from Japan SLC (Hamamatsu, Japan). The experimental protocols were approved by the Animal Use and Care Committee of the Tokyo Metropolitan Institute of Medical Science.

### 4.6. Stable Transfectants

MDCK, B16, COLO205 transfectants of HGS or its mutants truncated with various domains were established using the procedure as described previously [30]. To construct the expression vector encoding a wild-type HGS and its nine deletion mutants (PCQ, CQ, Q, ZP, Z, P, ZPC, PC, and C) tagged with Flag, we amplified with the following each sense and antisense primer with pME-HGS as a template. The Flag epitoped (DYLDDDDL) sense primers with *Eco*RI site were synthesized. Z sense primer: 5′-TTGAATTCATGGACTACAAGGACGACGATGA-CAAGATGGGGCGAGGCAGCGGCACC-3′, P sense primer: 5′-TTGAATTCATG-GACTACAAGGACGACGATGACAAGCCCCCAGAGTACCTGACCAGC-3′, C sense primer: 5′-TTGAATTCATGGACTACAAGGACGACGATGACAAGTTTA-GTGAGCAGTACCAGAAC-3′, Q sense primer: 5′-TTGAATTCATGGACACAAG-GACGACGATGACAAGCCCTTGCCTTATGCCCAGCTC-3′. The antisense primers with *Not*I site were synthesized. Z-antisense primer: 5′-ATAGTTTATGCG-GCCGCTAAGTGGTAGAGGCAGCTTT-3′, P-antisense primer: 5′-ATAGTTTATG-CGGCCGCTCAGGAAGTTATGGGCTGAGA-3′, C-antisense primer: 5′-ATAGTT-TATGCGGCCGCTAGGCACGCATCTGGACAGTCT-3′, Q antisense primer: 5′-AT-AGTTTATGCGGCCGCTCAGTCGAAGGAGATGAGCTGGGT-3′. Amplified DNA fragments were digested with *Eco*RI and *Not*I. The resultant fragments were ligated to pcDNA3 at the site of *Eco*RI and *Not*I. Each expression vector was sequenced to confirm the entire coding region sequence. MDCK, B16, and COLO205 cells cultured in a 100 mm diameter dishes were cotransfected with 30 µg of pcDNA3-FLAG-HGS deletion mutants and 1 µg of pSV2bsr (Funakoshi, Japan) in Opti-MEM (Gibco) using Lipofectamine2000 (Thermo Fisher Scientific, Waltham, MA, USA). Blasticidin S–resistant cells were selected at 5 µg/mL of Blasticidin S (Thermo Fisher Scientific, Waltham, MA, USA) in the medium and cloned by limiting dilution.

### 4.7. Immunocytochemical Analysis

Indirect immunofluorescence staining was performed as previously described [34] with minor modifications. Briefly, cells grown on a culture slide (BD Falcon, Franklin Lakes, NJ, USA) were fixed with 4% paraformaldehyde in phosphate-buffered saline (PBS, pH 7.4) for 20 min and permeabilized with 0.1% Triton X-100 in PBS for 20 min, followed by blocking with 5% bovine serum albumin and 0.5% (*v*/*v*) goat serum (Chemicon International Inc., Temecula, CA, USA) in PBS for 1 h at room temperature. The cells were then incubated with the primary antibodies for 1 h and washed in PBS three times for 15 min. The cells were next incubated for 1 h with the Alexa Fluor 488-conjugated goat F (ab’)_2_ fragment against either mouse IgG or mouse IgM. After being washed, the stained cells were examined under a BZ-X700 microscope (Keyence, Osaka, Japan).

### 4.8. Cell Culture in Soft Agar Medium

Trypsinized cells were resuspended (1 × 10^3^) in 1 mL of 2 × DMEM supplemented with 20% FBS, added to 1 mL of 0.6% agarose in DW (Koken, Japan), and seeded onto 35 mm dishes (BD Falcons) that had been pregelled with 2 mL of 0.6% agarose in medium. The cells in the dish were incubated at 37 °C for 2 h, after which 2 mL of RPMI-1640 medium supplemented with 10% FBS was added. The medium was changed every 3 days. The cells were observed at 14 days after the seeding.

### 4.9. Tumor Formation in Mouse

After a 2-week acclimation period, 8-week-old female C57BL/6 or nude mice were randomly divided into groups. Cells were resuspended at a cell density of 1 × 10^6^ cells in 100 µL of PBS and inoculated subcutaneously into the flank region of the mice. Tumor formation was monitored for 4 or 5 weeks after the inoculation by measuring the width (W) and length (L) of the tumors with W < L. The tumor volume was calculated according to the formula (W2 × L × π/6).

### 4.10. Cell Motility and Invasive Assay

Cell motility and invasive potential were assayed using biocoat culture inserts (Corning, Glendale, CA, USA) and Matrigel Inversion chambers (Corning, Glendale, CA, USA), respectively [35]. In brief, cells suspended in Opti-MEM supplemented with 0.1% (*w*/*v*) BSA were applied to the upper chamber, and Opti-MEM supplemented with 10% (*v*/*v*) FBS was filled into the lower chamber. The chambers were incubated for 24 h at 37 °C in the cell culture incubator, and then the non-transfer or non-invasive cells were removed from the upper surface of the membrane by scrubbing with a cotton-tipped swab. The cells on the lower surface of the membrane were fixed with 4% paraformaldehyde in phosphate-buffered saline (PBS, pH 7.4) for 20 min and permeabilized with 0.1% Triton X-100 in PBS for 20 min, followed by staining cell nuclei with propidium iodide (1 μg/mL) and RNase in PBS. Photographs of the nuclei-stained cells were taken under a BZ-X700 microscope and measured with NIH image software (ImageJ, ver. 1.52a).

### 4.11. SMAD and TCF/LEF Response Element Assay

Cells in 24-well plates were transiently transfected with 1 μg of Renilla luciferase reporter gene vector fused with a response element or no element and 0.1 μg of SV-firefly luciferase reporter gene vector as a control for transfection efficiency. pSmad RE-TK hRluc (F), pTCF/LEF RE-TK hRluc (F), TK hRluc, and pSV-firefly luciferase plasmid were purchased from RIKEN BioResource Center (Tsukuba, Japan) and Clontech, respectively. The activity of Renilla and firefly luciferase was determined in triplicate at 24 h after the transfection. Elemental activities were expressed as relative values using element activities.

### 4.12. In Vitro Anchorage-Independent/Dependent Growth Inhibition Test

An ultra-low attachment surface Corning Costar 96-well cell culture plate 3474 (Corning, Glendale, CA, USA) was used as an ultra-low attachment surface (ULAS) plate, and a tissue-culture-treated Corning Costar 96-well cell culture plate 3595 (Corning, Glendale, CA, USA) was used as a normal adhesion plate. COLO 205 cells (2 × 10^3^ cells/0.2 mL Opti-MEM medium, 5% FBS/well) were seeded in each well of the ULAS plate and normal adhesion plate. The oligopeptide OP12-462 were each added and cultured in a CO_2_ incubator at 37 °C.

After 7 days, 0.1 mL of Cell Counting Kit-8 solution (5-fold PBS diluted solution) was added, and the absorbance A450 nm was immediately measured and set as the T0 blank value. After incubating at 37 °C for 2 h, the absorbance A450 nm was measured again and set as T2. The absorbance difference between T2 and T0 was defined as the anchorage-independent or anchorage-dependent growth activity value.

### 4.13. Oligopeptide Tumor Growth Inhibition Test

The cells were resuspended at a cell density of 1 × 10^6^ cells in 100 µL of PBS and were inoculated subcutaneously into the flank region of 8-week-old female nude mice. The day when the tumor volume exceeded 250 mm^3^ was set as Day 0, and oligopeptide OP12-462 (50 mg/kg body weight) was administered as a 0.2 mL PBS solution in the tail vein once daily for 10 days from Day 0 to Day 9. Tumor sizes were measured daily.

### 4.14. Statistical Analysis

All statistical analyses were performed with EZR (version 2.7-1, Saitama Medical Center, Jichi Medical University, Saitama, Japan). The data are presented as the mean ± SEM, and statistical analysis was performed using a two-tailed Student’s *t*-test for independent samples, and a *p*-value less than 0.05 was considered statistically significant.

## Figures and Tables

**Table 1:** Morphology of HGS mutant cells with or without HGF stimulation.

HGS Mutants ^(a)^	Cell Morphology	
	HGF –	HGF + ^(b)^
MDCK - - - -	E ^(c)^	M ^(d)^
MDCK/HGS ZPCQ	M	M
- PCQ	M	M
- - CQ	M	M
- - - Q	E	M
ZP - -	E	M
Z - - -	E	M
- P - -	E	M
ZPC -	E	E
- PC -	E	E
- - C -	E	E

MDCK cells undergo morphological change from epithelial to mesenchymal cells through EMT induction by HGF stimulation. We established stable MDCK mutant cells overexpressing the HGS deletion mutants. ^(a)^ Z, P, C, and Q mean a zinc-finger, a proline-rich, a coiled-coil, and a proline–glutamine-rich domain of HGS, respectively. The morphology of MDCK mutant cells was observed without (HGF –) or with HGF (50 ng/mL) stimulation (^(b)^ HGF +). ^(c)^ E means an epithelial-like cell shape, ^(d)^ M means a mesenchymal-like cell shape.

**Table 2:** SMAD transcriptional activity in MDCK mutant cells.

MDCK Mutant Cells	TGF-β –	TGF-β + ^(a)^
MDCK cells	1.00 ± 0.016	11.1 ± 0.12
MDCK/HGS cells	566 ± 13.5	3664 ± 14.6
MDCK/C cells	0.91 ± 0.065	0.73 ± 0.088

Relative SMAD transcriptional activity downstream of the TGF-β signaling pathway was measured in MDCK, MDCK/HGS, and MDCK/C cells without (TGF-β –) or with TGF-β (50 ng/mL) stimulation (^(a)^ TGF-β +). Smad RE-TK hRluc, or TK hRluc, and SV-luc reporter gene plasmid were transiently transfected into the cells. Transcriptional activities of SMAD were calculated from Renilla luciferase activity and firefly luciferase activity.

## Data Availability

The original contributions presented in this study are included in the article. Further inquiries can be directed to the corresponding author(s).

## Abbreviations

BSA, bovine serum albumin; EMT, epithelial–mesenchymal transition; ESCRT, endosomal sorting complexes required for transport; HGF, hepatocyte growth factor; HGS, hepatocyte-growth-factor-regulated tyrosine kinase substrate; Kd, dissociation rate constant; MAb, monoclonal antibody; MDCK, Madin-Darby canine kidney; PBS, phosphate-buffered saline; SMAD, mothers against DPP homolog; TGF-β, transforming growth factor-β; Wnt, wingless-related integration site.
